# Numerical model for cough‐generated droplet dispersion on moving escalator with multiple passengers

**DOI:** 10.1111/ina.13131

**Published:** 2022-11-18

**Authors:** Ayato Takii, Masashi Yamakawa, Atsuhide Kitagawa, Tomoaki Watamura, Yongmann M. Chung, Minsuok Kim

**Affiliations:** ^1^ Department of Mechanical Engineering Kyoto Institute of Technology Kyoto Japan; ^2^ School of Engineering University of Warwick Coventry UK; ^3^ School of Mechanical, Electrical and Manufacturing Engineering Loughborough University Loughborough UK

**Keywords:** airflow, computational fluid dynamics, COVID‐19, pathogen transmission, risk of infection

## Abstract

To investigate the motion of virus‐laden droplets between moving passengers in line, we performed numerical simulations of the distribution of airborne droplets within a geometrically detailed model similar to an actual escalator. The left and right sides and the ceiling of the escalator model were surrounded by walls, assuming a subway used by many people every day with concern to virus‐laden droplets. Steps and handrails were incorporated in the model to faithfully compute the escalator‐specific flow field. The ascending and descending movements of the escalator were performed with 10 or 5 passengers standing at different boarding intervals. To resolve the unsteady airflow that is excited by a moving boundary consisting of passengers, steps, and handrails, the moving computational domain method based on the moving‐grid finite‐volume method was applied. On the basis of the consideration that the droplets were small enough, droplet dispersion was computed by solving the equation of virus‐laden droplet motion using a pre‐computed velocity field, in which the flow rate of a cough, diameter distribution, and evaporation of droplets are incorporated. The simulation resolved the detailed motion of droplets in flow, and therefore, we were able to evaluate the risk of viral adhesion to following passengers. As a result, we found that the ascending escalator had a higher risk of being exposed to virus‐laden droplets than the descending escalator. We also reported that the chance of viral droplet adhesion decreases as the distance from the infected person increases, emphasizing the importance of social distancing.

**Practical implication**

- Computational investigation of air flow provides unsteady behavior of the dispersion of virus‐laden cough‐generated droplets on an escalator, which consists of complex moving boundary conditions.
- Effects of traveling direction of an escalator and boarding position of passengers on the exposure risk are quantitatively examined and shows the exposure risk is higher for the ascending cases than the descending ones.
- Taking a farther distance from the infected person (social distancing) is important to reduce the risk of exposure.

## 1 INTRODUCTION

The pandemic caused by the novel coronavirus COVID‐19 is currently ongoing throughout the world, and careful measures are still required to curb significant loss of life and unprecedented economic loss. Fluid droplets expelled from infected individuals by respiratory events have attracted much attention in research because droplet transmission is considered to be one of the most critical mechanisms responsible for the rapid spread and continued circulation of infection among humans.^1^ According to a recent study, airborne transmission is the main route for the spread of COVID‐19.^2^ Multiple studies on saliva droplets have demonstrated that thousands of virus‐laden droplets were expelled from the mouth of an infected person through breathing, coughing, and sneezing.^3^, ^4^, ^5^ Bahl et al.^6^ used a high‐speed camera to monitor virus‐laden droplets expelled during coughing and sneezing. Asadi et al.^7^, ^8^ measured the rate of droplet release during human conversation and discussed the risk of spread. They demonstrated that viral droplets emitted during human speech could be significantly dispersed, and they discussed the potential spread of infection by asymptomatic patients. The basic mechanism of inhalation of particles in the air and adhesion of particles in airways was summarized by Inthavong.^9^

Focusing on the airborne infectious route, virus‐laden droplets expelled from the mouth or nose during coughing, sneezing and conversation have the potential to remain suspended in the air for a long time and travel a long distance. Khosronejad et al.^10^ carried out high‐fidelity computational fluid dynamics (CFD) simulations to elucidate the underlying physics of saliva particulate transport. They showed the following: finer particulates traveled farther, with 10‐μm particulates traveling the farthest away from the infected while large saliva particulates settled down soon after they were emitted; saliva evaporation increased the spreading distance. Wang et al.^11^ showed that 74% of droplet population remained in the air while the remaining either settled on the ground or completely evaporated within the coughing event (1 s), taking a realistic droplet size distribution and duration of a cough jet. Thus, it is important to understand how virus‐laden droplets spread from an infected individual to another.

Various computational models have been reported for numerical simulations of droplets and their airborne propagation routes. Yamakawa et al.^12^ performed a numerical simulation of airborne droplet dispersion over a long period of time using a classroom model with ventilation and showed that expelled droplets remaining suspended in the air affect the risk of infection. Yu et al.^13^ simulated the exposure of bioaerosols in offices equipped with mechanical ventilation and air conditioning systems. Thatiparti et al.^14^ performed a numerical simulation of cough aerosol dispersion in a mock airborne infection isolation room. Dbouk et al.^15^ investigated the effect of wind speed on the reach of salivary droplets produced by human coughing. This route may cause virus‐laden droplets excreted from coughing, sneezing, and during conversations to stay in the air for extended periods of time and travel long distances. There have been studies investigating droplet transmission in public transport. Yang et al.^16^ investigated the effect of cough jets on flow fields and droplet transport characteristics in an airliner cabin section. Talaat et al.^17^ studied aerosol transmission and intervention measures on a Boeing 737 cabin zone at different passenger capacities with and without sneeze guards. The effect of cough jet and ventilation was investigated in an elevator, which is considered to have a very high risk of infection due to it being a narrowly confined space.^18^, ^19^

During the pandemic, people tended to refrain from using elevators within a confined space to reduce infectious risk, preferring to use escalators within an open space. In particular, at stations and subways, the use of escalators is essential for traveling long height differences and many people use them with low‐risk awareness. However, the airborne transmission risk of escalators has not been fully understood; passengers can be very close together during busy hours such as commuting, long escalators installed at major terminal stations are semi‐opened with confined spaces on the left, right, and top. Moreover, considering dispersion characteristics of droplets expelled from an infected person that remain suspended for a long time, passengers may come into contact with virus‐laden droplets floating in a distant place as they move on the escalator. Therefore, it is important to predicate the movement of infectious droplets following passengers for a long time and understand the impact on distant passengers and appropriate boarding position. Li et al.^20^ investigated the effects of escalator slope and speed on the dispersion of cough‐generated droplets from a passenger in regard to steady flow with a Reynolds‐averaged Navier–Stokes solver. They observed the difference in wake structures behind the person to significantly impact droplet dispersion: these were defined as “downwash” for the ascending escalator, “upwash” for the descending one. The result showed that droplets expelled from an infected person on a descending escalator could travel long distances at the height of the person's head, and a person following behind was at risk. Wang et al.^21^ studied the dispersion of cough‐generated droplets from a person going upwards or downwards with a laboratory experiment in a water tunnel, using mannequin models similar to the one used by Li et al.^20^ They used a virtual particle simulation based on the velocity data from the experiments performed to study the effect of the initial position on particle concentration. The result showed that unsteady fluctuations of the airflow could result in the clustering of droplets, in contrast to droplet dispersion in a steady flow field. They suggested greater social distancing for people riding descending escalators and state that it is critical for a person on an escalator to cough or sneeze with their head down so that the dispersed droplets are entrained into the wake region toward the ground. In both studies, however, the effect of escalator geometry on airflow and droplet dispersion was not considered with a simple escalator model. Instead, the escalator was modeled as a moving floor and confined spaces around the escalator were not considered. This is very different from real escalator environments, where a complex flow field is generated by a dynamic combination of moving steps, moving handrails, and static side panels. Also, only a single passenger was included for risk evaluation of infectious droplets and the interaction with other passengers was ignored in the previous studies.^20^, ^21^

In this paper, we investigate the effect of cough‐generated droplets expelled from an infected person on other passengers riding the same escalator. A geometrically detailed escalator model is used with steps and handrails moving in ascending and descending directions.

## 2 NUMERICAL APPROACH

To solve this dynamical simulation, the flow fields are computed by solving the Navier–Stokes equations with the moving‐grid finite‐volume method. Next, virus‐laden droplets expelled by coughing are traced by solving a motion equation considering droplet evaporation and distribution of droplet size. Then, the risk of exposure to infectious droplets is quantitatively evaluated for different riding positions on the escalator.

### 2.1 Governing equations for fluid flow

In this study, the airflow in the escalator is computed first. The computed flow field is then used to track the movement of virus‐laden droplets. The continuity equation and the three‐dimensional uncompressed Navier–Stokes equations used to compute the airflow are as follows:

(1) $$\frac{\partial u}{\partial x}+\frac{\partial v}{\partial y}+\frac{\partial w}{\partial z}=0,$$

(2) $$\frac{\partial q}{\partial t}+\frac{\partial E}{\partial x}+\frac{\partial F}{\partial y}+\frac{\partial G}{\partial z}=0,$$

where
(3)

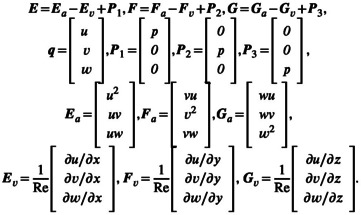




$E_{a},F_{a},G_{a}$ are advection vectors, $E_{v},F_{v},G_{v}$ are the corresponding viscous stress vectors, u,v,w are the corresponding velocity components in the *x*, *y*, *z* directions, respectively, p is pressure, and Re is the Reynolds number. Each variable is nondimensionalized as follows:

(4) x=x~L~0,y=y~L~0,z=z~L~0,t=t~L~0/U~0,p=p~ρ~aU~02,u=u~U~0,v=v~U~0,w=w~U~0,Re=ρ~aL~0U~0μ~a.

$\left(\sim \right)$ represents a dimensional quantity. ${\tilde{L}}_{0}$ is the characteristic length, ${\tilde{U}}_{0}$ is the characteristic velocity, ${\tilde{\rho }}_{a}$ is the density of the air (1.2 kg/m^3^), and ${\tilde{\mu }}_{a}$ is the viscosity of the air ($1.8\times 10^{-5}\mathrm{Pa}∙s$).

### 2.2 Discretization method for moving objects

The moving‐grid finite‐volume method,^22^ which is applied to unstructured grids, is used to solve the governing equations for computations with moving boundary conditions. Therefore, the entire computational domain moves along the escalator. In this method, the control volume is considered in four‐dimensional space (*x, y, z, t*) to satisfy the geometric conservation law for space and time. By applying the divergence theorem on the space–time unified control volume Ω, Equation (2) can be written as follows:

(5) $$\int_{\Omega }\left(\frac{\partial q}{\partial t}+\frac{\partial E}{\partial x}+\frac{\partial F}{\partial y}+\frac{\partial G}{\partial z}\right)d\Omega =\sum_{l=1}^{6}{\left(E{\tilde{n}}_{x}+F{\tilde{n}}_{y}+G{\tilde{n}}_{z}+q{\tilde{n}}_{t}\right)}_{l}S_{l}=0,$$

where $\tilde{n}=\left({\tilde{n}}_{x},{\tilde{n}}_{y},{\tilde{n}}_{z},{\tilde{n}}_{t}\right)$ is the outward unit normal vector in the space–time four‐dimensional space, l indicates the discretized planes of the control volume, and $S_{l}$ is the area of the plane. In the case of a tetrahedron cell, the control volume in the four‐dimensional space has six planes, where l=5,6 indicate the one perpendicular to the time *t*‐axis. Therefore, at l=5,6, ${\tilde{n}}_{x}={\tilde{n}}_{y}={\tilde{n}}_{z}=0$, and $S{\tilde{n}}_{t}$ equals to the volume V of the computational cell. The physical quantities are evaluated for l=5 at the time level m, for l=6 at the time level m+1, and or l=1,2,3,4 at the time level m+1/2. Finally, the governing equations are discretized as follows:

(6) $$q^{m+1}V^{m+1}-q^{m}V^{m}+{\left[\sum_{l=1}^{4}{\left(E{\tilde{n}}_{x}+F{\tilde{n}}_{y}+G{\tilde{n}}_{z}+q{\tilde{n}}_{t}\right)}_{l}S_{l}\right]}^{m+1/2}=0.$$

On the basis of this discretization, the moving computational domain (MCD) method^23^ is used to model the movement of the escalator, including that of the detailed components such as the steps, panels, and handrails, similar to an actual escalator. The physical quantities such as pressure and velocity are defined at the cell centers. The fractional step method^24^ is used to solve these equations. The Lower‐Upper Symmetric‐Gauss–Seidel (LUSGS) method^25^ is used to determine the pseudo‐time stepping of the equations for velocity. The Poisson equation for pressure is solved using the Bi‐Conjugate Gradient Stabilized (Bi‐CGSTAB) method.^26^ The in‐house code was used to simulate the various moving objects.^27^, ^28^, ^29^ The quantitative validation is also presented in Appendix A in Appendix S1.

### 2.3 Motion equations for airborne virus‐laden droplet

In this paper, the motion of airborne droplets was simulated using one‐way coupling, and the effect of droplet movement on the flow field was ignored due to very small virus‐laden droplets. The droplets expelled from the mouth of a passenger consist of saliva. In addition, it is assumed that all droplets are spherical and their interactions with each other are negligible. The equation of droplet motion is defined as follows:

(7) $$\frac{d\rho_{d}Vq_{d}}{\mathrm{dt}}+\rho_{d}Vg+\frac{C_{D}\rho_{a}\pi r^{2}\left|q_{d}-q_{a}\right|\left(q_{d}-q_{a}\right)}{2}=0,$$

where, $\rho_{d}$ is the density (998 kg/m^3^), V is volume, r is radius, $q_{d}$ is velocity vector of the droplet, and g is the gravitational acceleration vector. $q_{a}$ is the airflow velocity at the center position of the droplet, which has second‐order accuracy based on gradient reconstruction in a computational cell. Here, considering the effect of contaminants in saliva such as proteins, the boundary between air and water is treated as a no‐slip condition.^30^ As a rigid sphere, the following equation is used for the drag coefficient^31^:

(8) $$C_{D}=\frac{24}{Re_{d}}\left(1+0.15Re_{d}^{0.687}\right),$$

(9) $$Re_{d}=\frac{2r\left|q_{d}-q_{a}\right|}{\nu_{a}}.$$

$Re_{d}$ is the Reynolds number based on a droplet and $\nu_{a}$ is the kinematic viscosity coefficient of the air. The time change of the droplet radius due to evaporation is estimated by the following equation from the Clausius–Clapeyron relationship^32^:

(10) $$\frac{\mathrm{dr}}{\mathrm{dt}}=\frac{D\left(\mathrm{RH}/100-1\right)e_{s}}{\rho_{d}R_{v}\mathrm{Tr}},$$

where t is time, D is diffusion coefficient of water vapor, *RH* is relative humidity (60%), $R_{v}$ is gas constant of water vapor, and *T* is temperature (27°C). The saturated vapor pressure, $e_{s}$, can be calculated as

(11) $$e_{s}\left(T\right)=e_{s}\left(T_{0}\right)\times \exp\left[\frac{L\left(T-T_{0}\right)}{R_{v}TT_{0}}\right].$$

where $T_{0}$ is base temperature (0°C) and L is the latent heat of vaporization of water. The saturated vapor pressure at the temperature of $T_{0}=0$ is $e_{s}\left(0\right)=6.11\times 10^{2}\mathrm{Pa}$. In this paper, the minimum radius is set as the equilibrium state in which the droplets do not evaporate any more. The ratio of the equilibrium radius to the initial radius of the droplets is reported by Marr et al.^33^ as a function of the humidity.

### 2.4 Model geometry and simulation conditions

**FIGURE 1:**
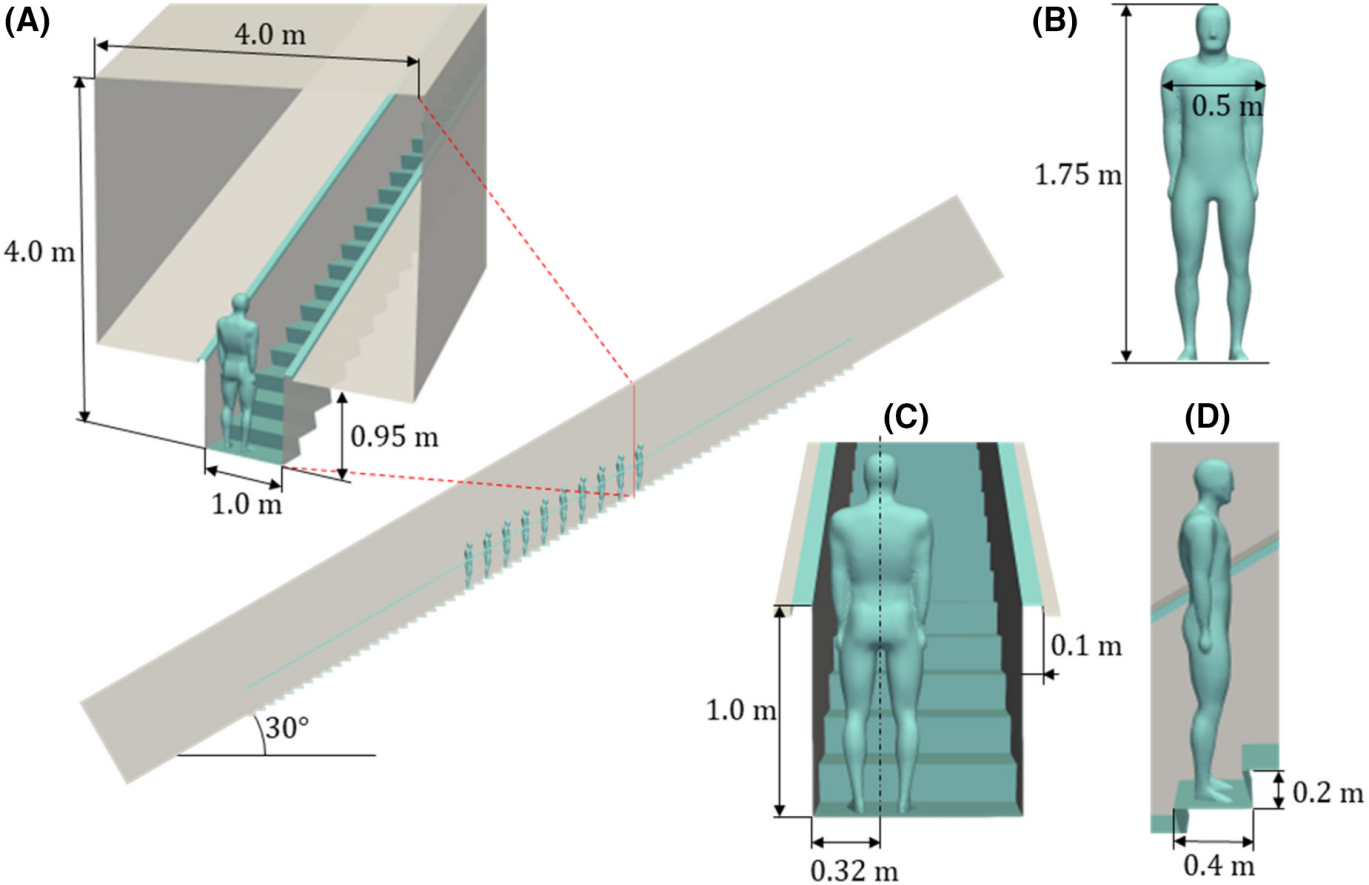
Illustration of escalator model geometry: (A) cross‐sectional view of escalator corridor, (B) passenger, (C) handrails and position of passenger, and (D) step depth and height. Moving boundary condition is applied to blue‐colored walls composed of steps, handrails, and passengers.

**FIGURE 2:**
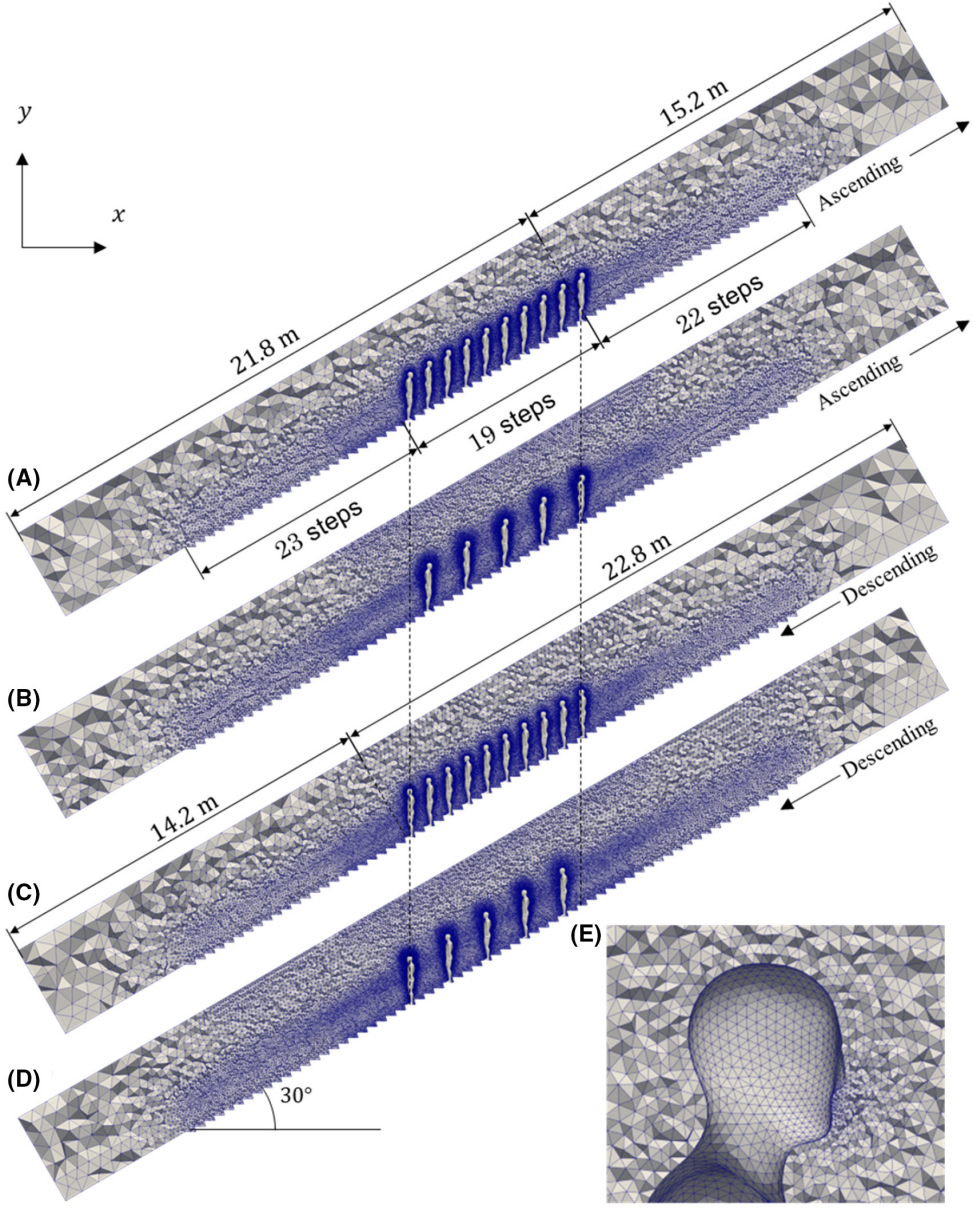
Appearances of computational grids for (A) ascending movement with 10 passengers (CASE I), (B) ascending movement with 10 passengers (CASE II), (C) descending movement with 5 passengers (CASE III), and (D) descending movement with 5 passengers (CASE IV). (E) Close‐up grids around passenger's head.

In this paper, a detailed escalator model is used for numerical simulations. The angle of inclination of the escalator is 30 degrees. On the basis of general specifications,^34^ the escalator moves at constant speed 0.5 m/s. Figure 1 shows the escalator model geometry: (a) the vertical cross‐section dimensions of the escalator corridor are 4.0 m × 4.0 m (W × H) at the tip of steps and the step width of the escalator is 1.0 m; (b) the model of an adult passenger with a height of 1.75 m and a shoulder‐width of 0.5 m; (c) handrails are 1.0 m high, 0.1 m wide, and 0.05 m thick. The passengers are standing on the left‐hand side of the escalator, and the midplane of the passenger is positioned 0.32 m from the left panel in the traveling direction; (d) a step has a depth of 0.4 m and a height of 0.2 m. Steps, handrails, and passengers (colored blue in the figure) are moving in the simulation with the moving boundary condition as in a real escalator. Four numerical computations are carried out: moving in both upwards and downwards directions with 5 and 10 passengers, as summarized in Table 1. Figure 2 shows the computational grids for (a) ascending with 10 passengers (CASE I), (b) ascending with 5 passengers (CASE II), (c) descending with 10 passengers (CASE III), and (d) descending with 5 passengers (CASE IV). Figure 2E shows a detailed view of grids around the passenger head. The generated grids have a minimum grid size of ~2 mm on the mouth surfaces where a cough jet is exhaled.

**TABLE 1:** Computational conditions

CASE	Moving direction	Number of passengers	Distance between passengers, *Δ*(m)	Coughing direction
I	Ascending	10	0.69	Frontward
II	Ascending	5	1.4	Frontward
III	Descending	10	0.69	Frontward
IV	Descending	5	1.4	Frontward
I‐L	Ascending	10	0.69	45 degrees to left

**FIGURE 3:**
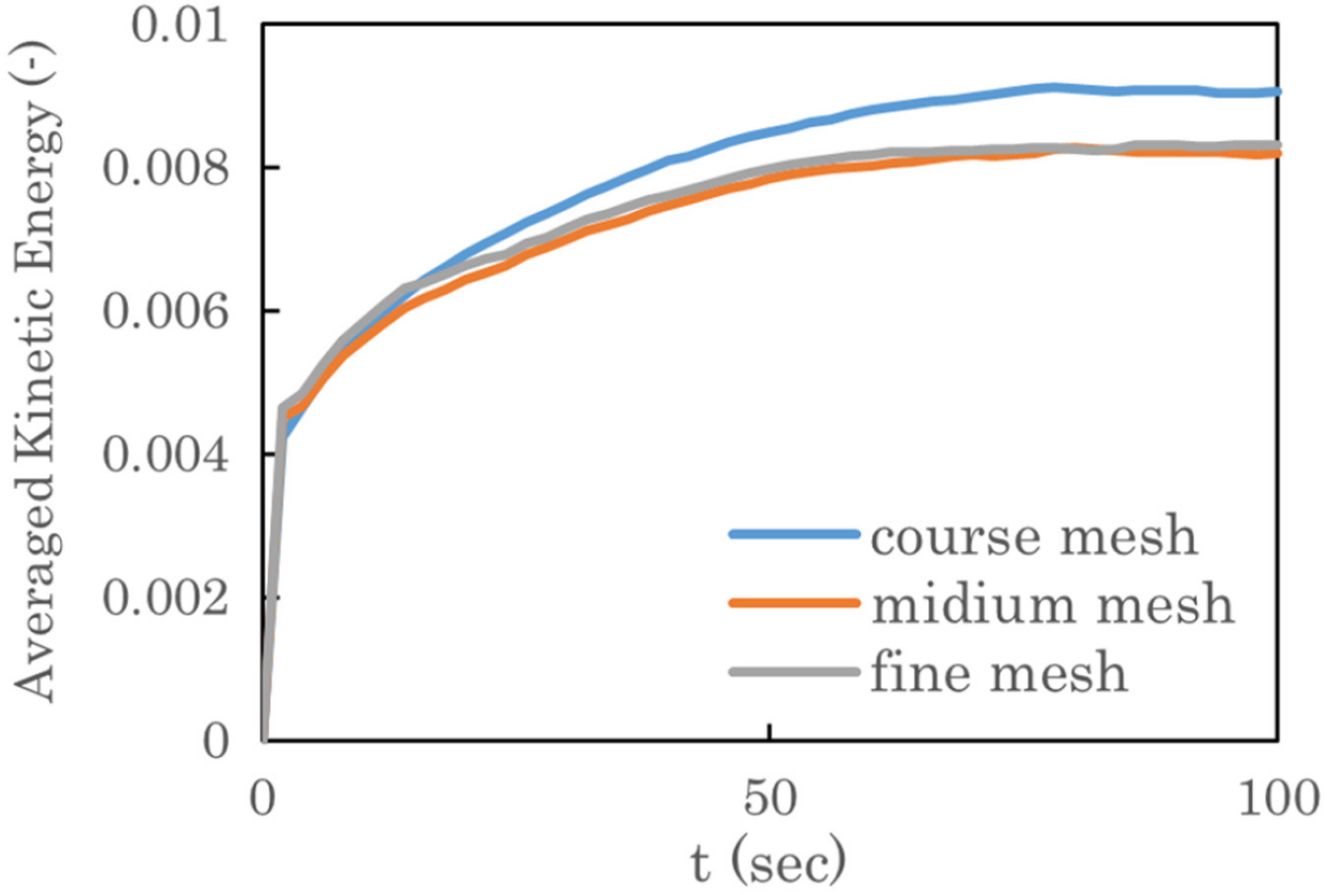
Time variation of spatially averaged kinetic‐energy.

To ensure that the obtained results are independent of the grid resolution, the effect of the grid size is preliminarily tested with a mesh spacing of ~6 mm (coarse), ~2 mm (medium), and ~ 1 mm (fine) on the mouth surfaces. As described in Figure 3, we confirmed that the spatially averaged kinetic‐energy does not vary significantly between the medium and fine meshes. The difference in the well‐developed state was smaller than 2%, even though the resolution differs by a factor of 2. Therefore, we use the medium mesh in this study. We have also conducted the quantitative validation by considering the flow around a sphere at Re = 100, which is a well‐known configuration, and confirmed that the present method can achieve to simulate the flow at moderate Reynolds number. Details are provided in Appendix A and Figure A1 in Appendix S1. Notably, owing to the presence of boarding passengers, the shape and number of grids are slightly changed. The total number of grid cells used in the four cases are approximately (a) $3.6\times 10^{6}$, (b) $4.5\times 10^{6}$, (c) $3.5\times 10^{6},$ and (d) $4.8\times 10^{6}$, respectively. The computational domain has a total length of 37 m and includes 64 steps of the escalator. For computational stability, the front and rear ends of the escalator are modeled as simple slopes to prevent computational divergence owing to unsteady flow generated by steps on the boundary surfaces. Passengers line up behind each other on the steps by skipping one step (a horizontal distance of 0.69 m) in the case of 10 passengers and three steps (a horizontal distance of 1.4 m) in the case of 5 passengers. In this study, the leading passenger is an infected person who expels virus‐laden droplets from their mouth by coughing. The shape of the mouth is modeled as an ellipse of 37 mm wide and 17 mm high.

We use a one‐way coupling simulation; airflows are solved using the moving‐grid finite‐volume method (explained in § 2.1) and virus‐laden droplet motions are solved considering the effect of spherical particle drag, gravity, and evaporation (explained in § 2.3). The escalator starts moving from a stationary state with initial conditions of zero air velocity and pressure. For boundary conditions, the upstream boundary in the traveling direction is set to the inflow boundary: velocity is zero and pressure is the Neumann condition. The downstream boundary is set to the outflow boundary: velocity is the Neumann condition and pressure is zero. Moving components composed of passengers, steps, and handrails have non‐slip wall conditions with the same velocity as the escalator speed as well as the Neumann condition for pressure. The other boundaries are non‐slip static walls where velocity is zero and pressure is the Neumann condition. The Reynolds number based on the escalator speed and step width is Re ≈ 3 × 10^4^. The time step of fluid computation is 1 × 10^−4^ s during coughing and 2 × 10^−3^ s otherwise. The threshold for convergence of velocity and pressure is 0.01% of the initial residual in the pseudo‐time stepping.

**FIGURE 4:**
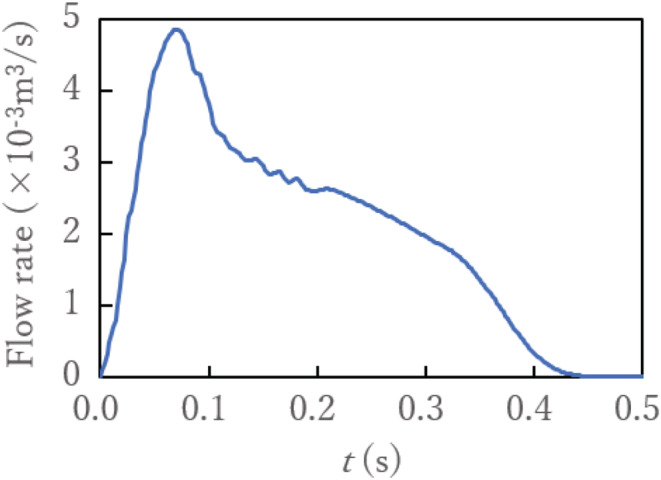
Coughing flow rate expelled from passenger's mouth.

**FIGURE 5:**
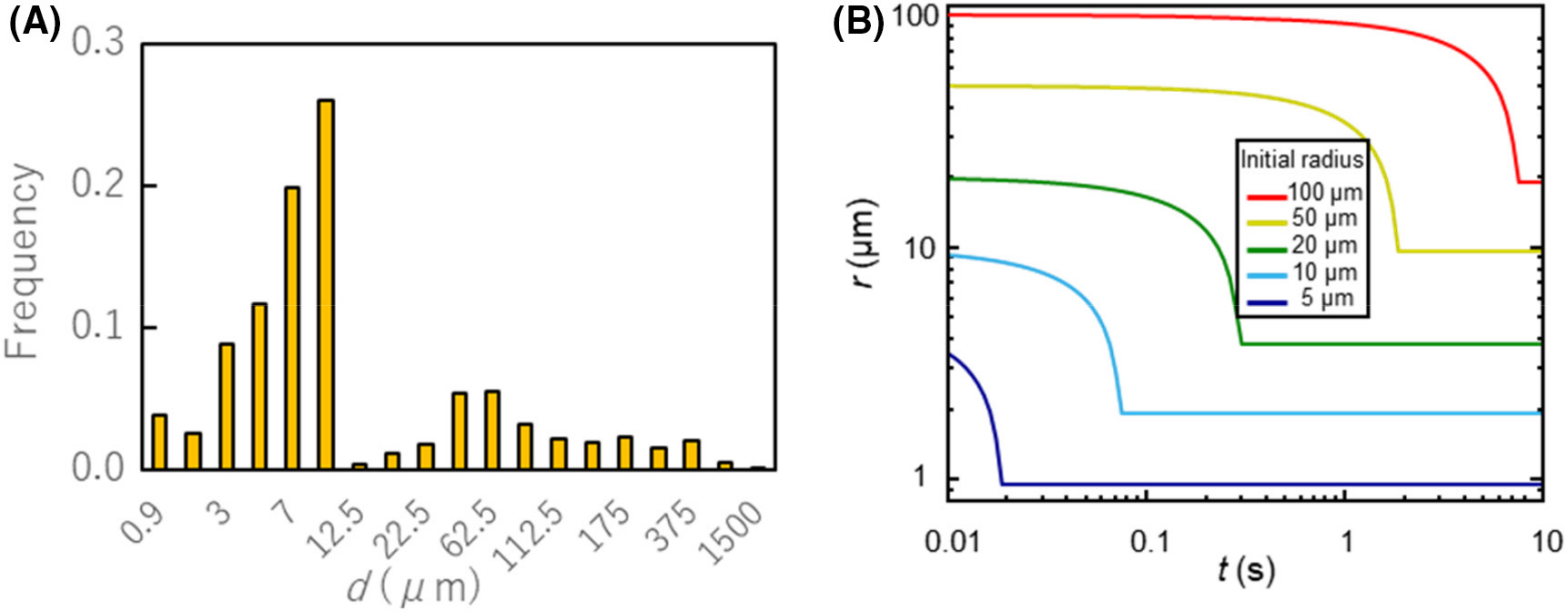
(A) Frequency distribution of droplet diameters expelled from passenger's mouth by a cough. (B) Time variation of droplet radius for each initial size expelled by cough. Considering evaporation and minimum limit, droplet radius decreases to 19% of initial size.

The leading person coughs once after 20 s. To simulate the cough jet exhaled from the mouth, the flow rate measured from a spirometer test is used as a boundary condition. Figure 4 shows the flow rate of the cough ejected from the passenger's mouth.^35^ Human respiratory events produce droplets with typical sizes in the range of 10^−1^ μm–10^3^ μm.^4^, ^7^, ^36^, ^37^ This wide range of droplet sizes results in complex particle patterns in the cough flow. To investigate realistic droplet motions, we use the frequency distribution of the diameter of the droplets expelled from the mouth by coughing shown in Figure 5A. This particle distribution was used in the authors' previous study.^12^ Figure 5B shows the time variation of cough‐generated droplet radius *r* based on Equation (10) with a minimum radius. The total number of droplet particles expelled from the mouth in a cough is 10 900.^12^ The number of droplets generated per unit time is proportional to the flow rate. These droplets are uniformly distributed on the mouth surface of the infected person as the initial location. The initial velocity is set to be the same as the moving velocity of the escalator.

Next, to quantitatively assess the risk of exposure to infectious droplets, we measure the number of droplets adhered to the head and body of each passenger, where the droplets that penetrate surfaces are considered to be adhered. In this risk evaluation, apart from the droplet dispersion simulation, the number of droplets released by coughing is set to be 100 times larger (i.e., 1 090 000 droplets) to achieve better accuracy. The effect of the riding position (i.e., distance from the infected passenger *Δ*) on the risk is investigated.

### 2.5 Estimation of infection probability

To estimate the risk of infection quantitatively, we adopt the dose–response model used by Bale et al.^38^ In the model, the probability of infection is written as follows:

(12) $$\Phi =1-e^{\left(-\alpha \frac{N}{N_{0}}\right)},$$

where α is a factor that accounts for the effect of the variant strain or vaccination, N is the total number of virions inhaled, and $N_{0}$ is the number of viral particles leading to infection. In this study, we choose α=1, even though the emergence of highly infectious strains can significantly increase the probability of infection. N and $N_{0}$ must be estimated to compute the probability of infection *Φ*. Although the reported values vary by approximately an order of 100–1000, we set $N_{0}$ to the value of 900 in this study. The droplets attached to a passenger's body are considered to present the amount of exposure to viral particles. N is estimated by following equation:

(13) $$N=\lambda_{v}v_{d}^{0}$$

where $\lambda_{v}$ is the viral load or viral density (copies/m^3^), and $v_{d}^{0}$ is the total volume of droplets attached to the passenger's body. We set $\lambda_{v}$ = 7 × 10^6^ copies/ml, as used in the literature. $v_{d}^{0}$ is computed by using the droplet size at the time of ejection from the mouth of infected person because the viral load contained in the droplet does not vary during evaporation.

## 3 RESULTS AND DISCUSSION

### 3.1 Air flow around passengers

**FIGURE 6:**
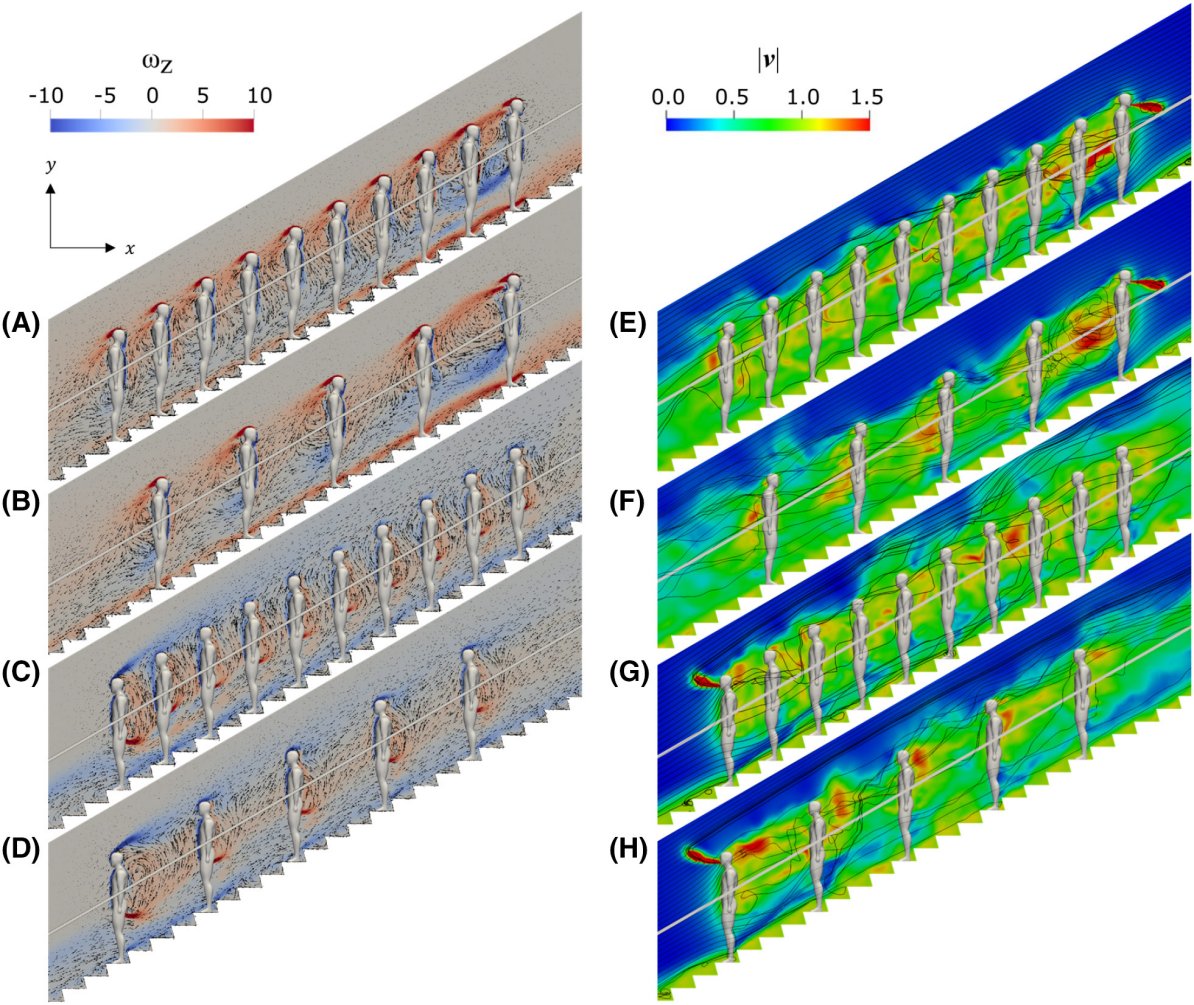
Time‐averaged flow fields (A–D) from *t* = 0 s–80 s with superimposition of the *z*‐component contours of vorticity and relative velocity vectors on the vertical plane including midline of passengers, and instantaneous flow fields (E–H) with superimposition of the contours of velocity magnitude and streamlines at *t* = 0.4 s after coughing start time.

Flow fields around passengers on a moving escalator are shown in Figures 6A–D as time‐averaged over 80 s without cough, and Figures 6E–H as instantaneous flow fields at 0.4 s after the coughing. For the ascending case with 10 passengers (see Figures 6A–E, CASE I), the flow separates at the head and reattaches at the back of each passenger. This is similar to the experimental results of Wang et al.^21^ although only a single passenger was considered in their water tunnel experiment. Note that some flow separates from the head and reattaches to the passenger behind. For the first two passengers, the flow separation occurs at the hip due to the flow passing through the inseam and reattaching at the back, generating a recirculation region. At the boarding positions of the third and subsequent passengers, downwash toward the steps is observed in the spaces between passengers, and flow separations at the hip are no longer observed. The steps generate a stronger flow in the direction of travel of the escalator than the simple sloping floor used in experiments.^20^, ^21^ The flows separate at the tip of the steps, and the magnitude of recirculation (vorticity) decreases as the distance from the first passenger increases due to an augmented flow entrainment. For 5 passengers with a wider riding distance (see Figures 6B,F), in addition to the flow separation at the head, the flow separates at the hip and reattaches at the back; however, the magnitude of vorticity decreases toward the passenger at the back of the queue. A large recirculation is formed in the wider distance between passengers due to a reduced downwash effect. For the descending case with 10 passengers (see Figures 6C,G), flow separates at the hip and reattaches at the head while the separated flow from the head flows backward. This is similar to the experimental result in Wang et al.^21^ For the descending escalator (CASE III and IV), in the spaces between passengers, upwash blowing is observed. As a result, the flow separates from the head and advects over the heads at the further back position. For 5 passengers (see Figures 6D,H), a large recirculation region is observed behind the first passenger.

### 3.2 Dispersion of virus‐laden droplets

**FIGURE 7:**
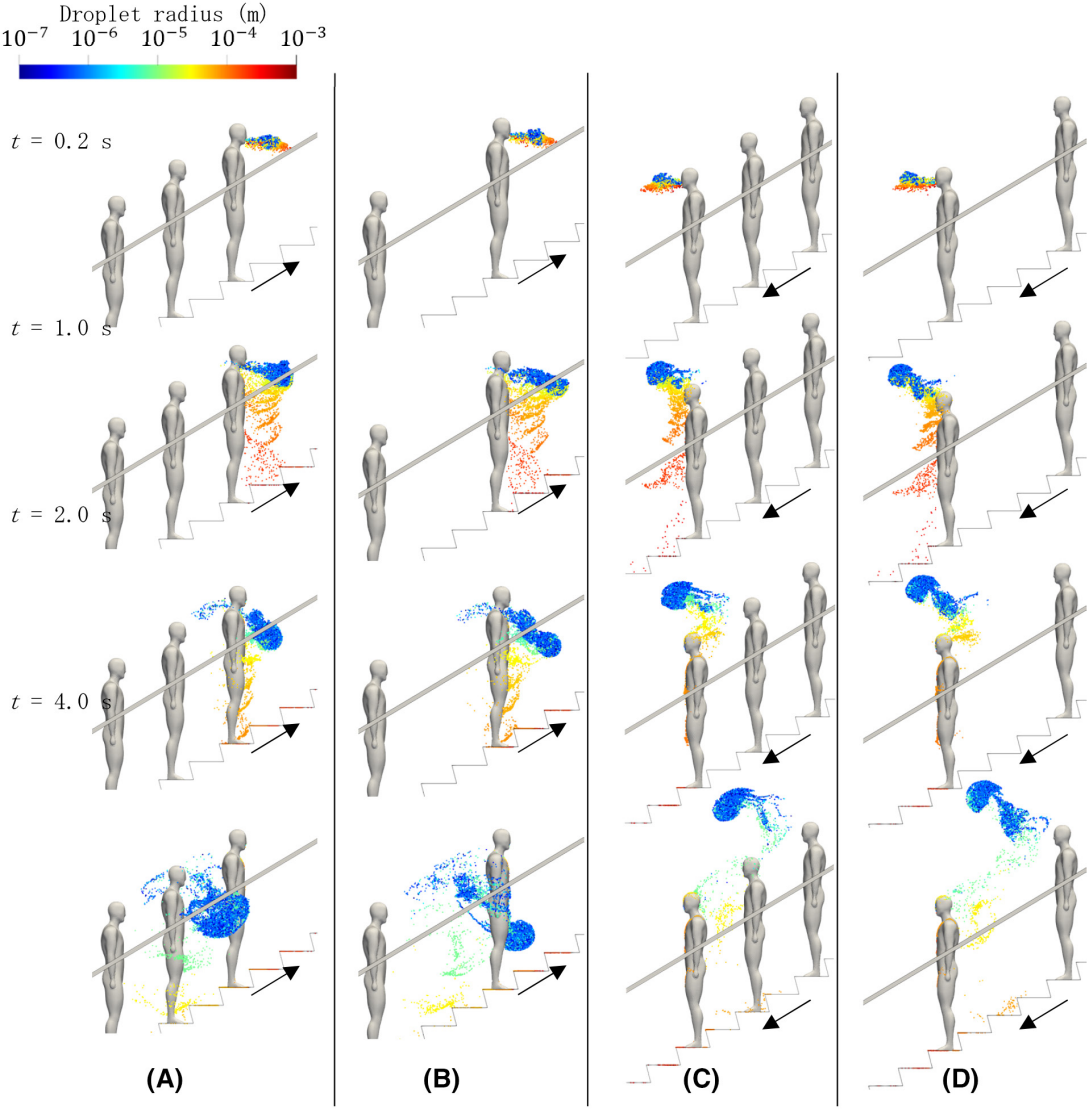
Dispersion of virus‐laden airborne droplets expelled from leading person (infected) at *t* = 0.2, 1.0, 2.0, 4.0 s immediately after coughing for the following cases: ascending with (A) 10 and (B) 5 passengers, and descending with (C) 10 and (D) 5 passengers. Droplets are colored by radius.

**FIGURE 8:**
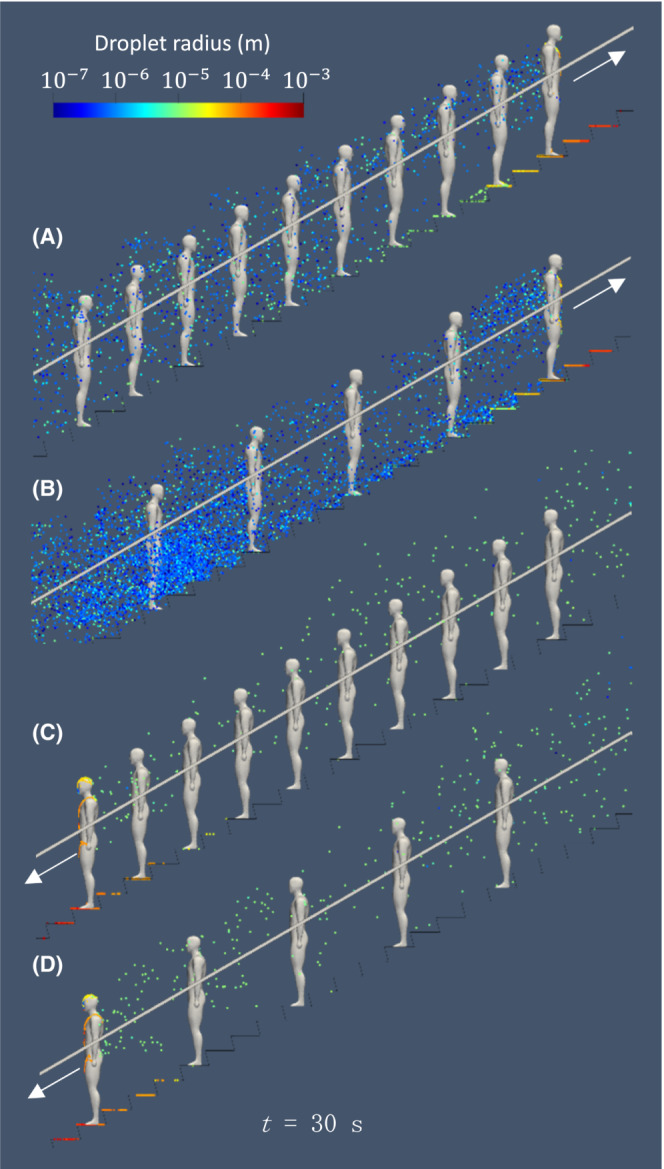
Instantaneous dispersion of virus‐laden airborne droplets around passengers 30 s after coughing for the following cases: ascending with (A) 10 and (B) 5 passengers, and descending with (C) 10 and (D) 5 passengers. Droplets (colored by radius) dispersed downward remain suspended around passengers for a long time, following escalator movement.

After the escalator moved for 20 s to ensure the fully developed flow around the passengers, the leading passenger ejected droplets from their mouth by coughing in the horizontal direction. Figure 7 shows the temporal distribution of airborne droplets at *t* = 0.2, 1.0, 2.0, 4.0 s after coughing for all four cases. Droplets are colored by their radii: red indicates large droplets, while blue represents small droplets. A strong jet is formed due to the coughing, and droplets emanate from the mouth. The jet strength (at *t* = 0.2 s) does not appear to be affected by the direction of movement. Once the coughing is completed, the jet starts to form a vortex ring,^39^ and this is more prominent in the downwards movement (Figure 7C,D). As expected, large droplets fall faster than small ones due to the gravity and inertia force from flow fields in accordance with Equation (7). Note that the discontinuous dispersion of falling droplets is due to the discrete distribution of initial droplet size used in this simulation (see Figure 5). At time *t* = 2.0–4.0 s, the effect of evaporation of droplets on dispersion is observed; a number of droplets stopped falling as their radii decrease over time while all droplets moved downwards in the quiescent fluid due to gravity. In the ascending cases (see Figure 7A,B, CASE I and II), droplets are located near the waist of the first passenger, and the droplet radius decreases (their colors vary from yellow to green). For small particles, as the drag force is dominant compared with gravity, droplet motion is likely to follow the airflow. For this reason, airflow strongly affects the droplet motion and prevents small particles from settling down. On the contrary, in the descending cases (see Figure 7C,D, CASE III and IV), most of the droplets expelled from the infected passenger are blown upward and entrained into a vortex ring traveling over the heads of passengers. Figure 8 shows the droplet dispersion 30 s after the coughing. In the ascending cases, small (*r* < 10^−6^) and medium (10^−6^ < *r* < 10^−4^) size droplets remain suspended around the passengers. Note that in the ascending case with 5 passengers, a large number of droplets are still moving with passengers. This is because just after coughing, droplets move downward into steps where the air flows in the same direction as the passengers' traveling due to stirring by the convex shape of the step. On the contrary, only medium‐sized droplets float in the descending case because small droplets are entrained into a vortex ring and passed over the passengers' heads. Therefore, the suspended height of cough‐generated droplets has an impact on long‐term exposure time of airborne droplets to passengers.

### 3.3 Risk evaluation by droplets adhesion to passengers

**FIGURE 9:**
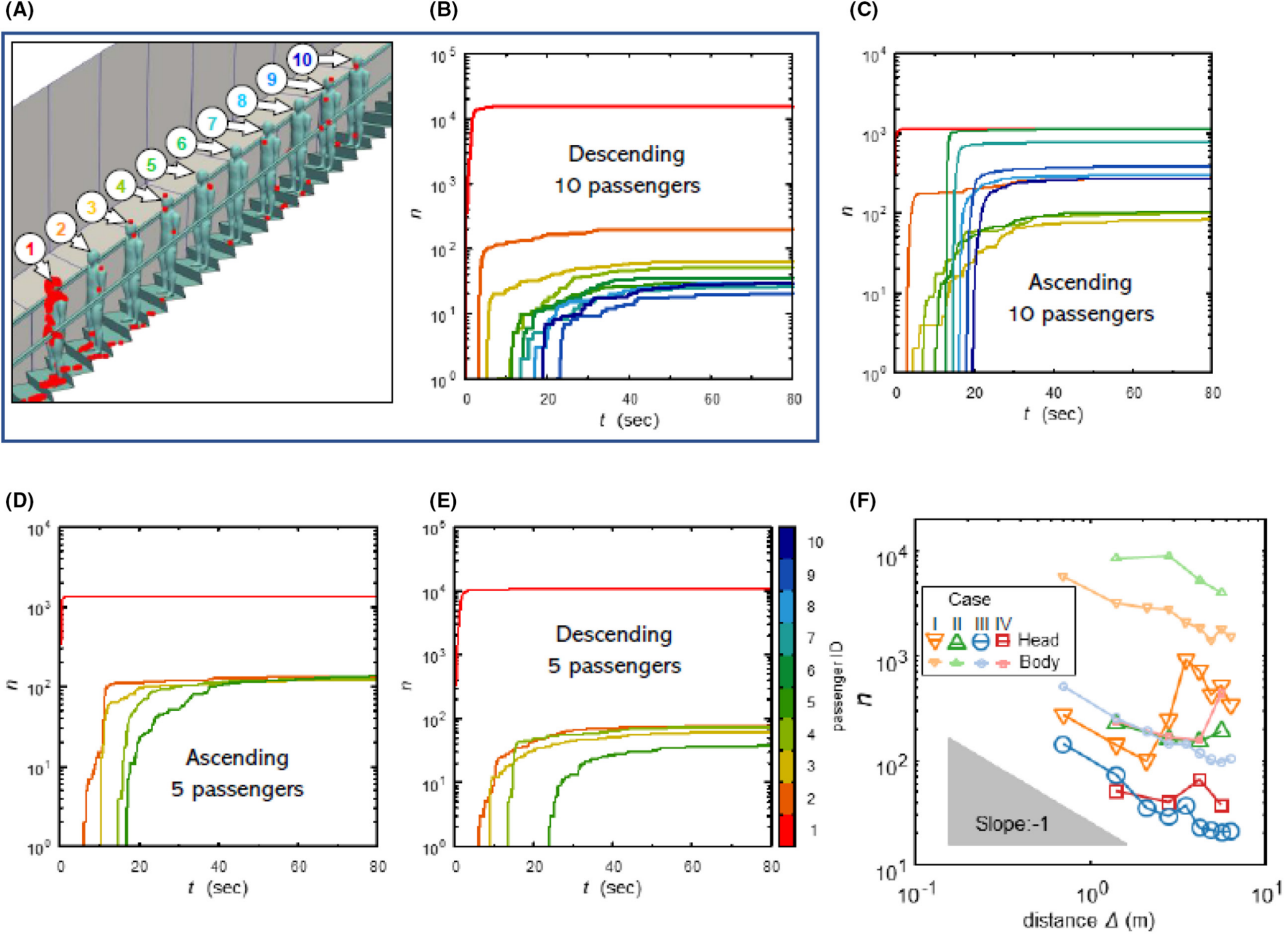
Impact of boarding position on viral adhesion. (A) and (B) are typical result in four cases: (A) appearance of droplet (red particle) adhesion, (B) time evolution of the number of droplets attached to passengers' heads in logarithmic scale. The order of droplet adhesion amount differs depending on the distance from the infected person. (C–E) are other results (CASE I, III, IV). (F) Relationship between horizontal distance from infected person and the number of droplets attached to following passengers. The amount of droplet adhesion is the ensemble average of computations with different cough timing.

Figure 9A shows a typical snapshot of droplets attached to passengers (CASE III). From the figure, the number of attached droplets decreases as the distance from the infected person (passenger ID 1) increases. Figure 9B shows the temporal evolution of the number of attached droplets for CASE III; the number significantly depends on the distance from the infected person and varies in orders of magnitude. The number of attached droplets increases with time but reached a plateau when *t* > 40 s. Figure 9C–E display the temporal evolution of the number of attached droplets in CASE I, III, and IV, respectively. In the ascending cases, CASE I (10 passengers) has a higher risk of contact with droplets than CASE II (5 passengers), whereas people in the latter case are exposed to droplets for a long time due to the large recirculation area and stagnated flow near steps. The possible difference between CASE I and CASE II is the height of the high concentration of droplets; the droplet cloud remains suspended around the area above the waist of the coughing person with 10 passengers but falls into the inseam of the coughing person with 5 passengers. In other words, the risk for passengers increases mainly due to the interaction with a high‐concentrated droplet cloud rather than prolonged exposure to droplets entrained by recirculation and stagnation near steps. For the descending cases, no significant effect of riding distance is found although CASE IV (5 passengers) has a lower exposure risk than CASE III (10 passengers).

Next, we compare the risk of contact with virus‐laden droplets between the ascending and descending cases: the descending cases have a lower risk than the ascending ones because most droplets expelled from the mouth entrained into a vortex ring and passed over the heads of passengers due to the upwash, as shown in Figure 7. The growth pattern of exposure risk over time is different between the ascending and descending cases; the number of attached droplets rapidly increases for the ascending cases, whereas it gradually increases for the descending ones. This results from an interaction between passengers and the high‐concentrated droplet cloud. The present CFD study shows that the risk of exposure to virus‐laden droplets is higher in the ascending cases than descending ones, where virus‐laden droplets are blown up by upwash and wider riding distance reduces the risk. This finding is the opposite of the observations of Li et al. and Wang et al. Note that the elevator models used in the experimental studies^20^, ^21^ were different from that used in the present study. While a realistic elevator model was used in this CFD study, railing and steps were not included in the experiments. First, the steps increase floating time of airborne droplets near the floor. However, the major factor in the increase in droplet exposure is contact with high‐concentration droplet clouds. Early droplet clouds are greatly affected by the intensity and expelled angle of a cough. In this study, a direct factor in the low risk for “descending escalators” is that the droplets expelled farther forward (upward) by a cough and did not descend. The cough condition was a steady flow of 6.5 m/s in the previous studies while that in this study is an unsteady flow instantaneously expelled at a maximum of ~10 m/s. Therefore, it is considered that the droplets are transported far away by the vortex ring. Second, the left and right sides of the passenger's lower body are surrounded by handrails, which is thought to encourage the generation of updrafts rather than open spaces. These may contribute to the opposite trend. Finally, the fundamental difference is that previous studies used a single passenger model for the fluid computation, assessing the risk to a passenger behind the fictitious location. In other words, wake interference was not computed. In addition, the risk was assessed by observing the floating position of the airborne droplets and there was no discussion of contact between the droplets and a passenger behind the infected passenger.

Next, we quantitatively estimate the risk of infection using the dose–response model, focusing on the second and last passengers in CASE I, which is the highest‐risk case. In this study, the number of droplets and the total volume attached to the passenger at 80 s after the onset of coughing are as follows: 108 and 1.9 × 10^−10^ m^3^ for the second passenger; 2 and 4.3 × 10^−13^ m^3^ for the last passenger. The numbers of viral particles leading to infection reported by various studies are different. Table 2 lists the probabilities of infection computed for $N_{0}$ = 100, 500, 1000, and 2000. Although the estimated value of $N_{0}$ changes the probability of infection by an order of magnitude, we find that the second passenger is approximately 25–40 times more likely to be infected than the last passenger.

**TABLE 2:** Probability of infection *Φ* for CASE I

Boarding position	Number of viral particles leading to infection $N_{0}$			
	100	500	1000	2000
Second	74%	24%	13%	6.5%
Last	3.0%	0.6%	0.3%	0.2%

Considering unsteady flow fields, simulations of droplet dispersion were carried out with several different cough timings (20, 30, 60 s after escalator starting time). Although the risk changed somewhat due to the unsteady fluid motion, a similar trend is observed from all simulations with different cough timings. Figure 9F shows the relationship between the horizontal distance from the infected person to following passengers and the number of droplets attached to the passengers on a head and body (entire surface of a passenger excluding their head). The amount of droplet adhesion shows the ensemble average of computations with different cough timings. Although there are a number of variations, the amount of droplet adhesion decreases as the distance from the infected person increases. This trend shows the importance of physical distancing as the World Health Organization recommends.^40^ Here, the slope of the graph is almost −1, which means doubling the distance from the infected person reduces the amount of droplet adhesion by half. This relationship is analogous to the stationary solution of the diffusion equation in spherical coordinates, even though a droplet is not a continuum and its motion is discretely computed herein. This scaling law will be a key consideration to assess the risk of viral droplet contact. However, as shown in Figure 9F, under a number of cases, in particular, ascending with 10 passengers (CASE I), being positioned at a farther distance away from the infected person could present a higher risk than at a closer one. This shows that the variation of exposure risk depends on complex fluid dynamics, unlike the result of a simple diffusion phenomenon.

**FIGURE 10:**
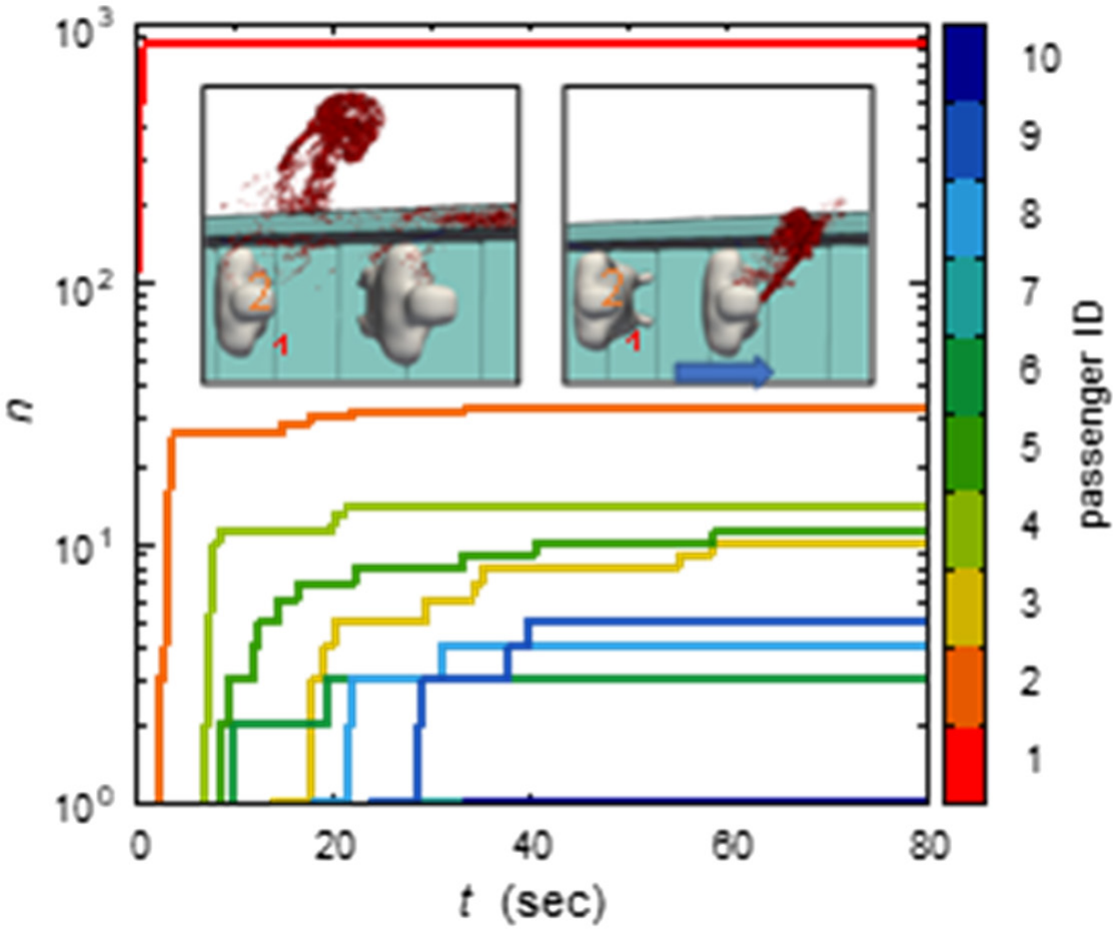
Time evolution of viral droplet adhesion in ascending escalator with 10 passengers under coughing leftward by 45 degrees. Insets show snapshots around first and second passengers at *t* = (left) 2.3 s and (right) 0.3 s after cough.

Finally, further simulations were performed to examine the effect of the coughing direction: the same configuration as CASE I were used but the angle of the cough was set at 45 degrees to the left (outside of the escalator). Under this condition, droplets travel outside of the escalator and further away from the passengers (see Figure 10), reducing exposure risk by 90% (only 1/10 of CASE I). However, the handrail of the escalator can be contaminated with a large number of droplets. This result indicates the importance of practicing cough‐etiquette (direction toward a place where there are no people) and handwashing (after touching a handrail) in daily‐life to prevent infection of COVID‐19, influenza, etc.

## 4 CONCLUSION

This paper investigated the dispersion of virus‐laden droplets expelled from an infected person on ascending and descending escalators with 10 and 5 passengers using CFD under an isothermal condition. To simulate characteristic flow on the escalator, detailed geometry components such as steps, panels, and handrails were incorporated into the computational model. As a result of moving the escalator, the following flows were obtained around passengers: ascending, downwash blowing down toward steps; descending, upwash flowing upward. Wider spacing of passenger reduced the effect of upwash and downwash; large recirculation regions were formed as the wake of passengers. In the vicinity of steps, flows stagnate in a concave region and were entrained to follow the movement of the escalator. Although these flows were computed without using a turbulence model, vortex rings were resolved by dynamical computation, which was an important factor affecting the chance of contact with the virus‐laden droplets.

Using these flow fields, airborne transmission of virus‐laden droplets was simulated with one‐way coupling by solving the motion equations, where the flow rate of a cough, diameter distribution, and evaporation of droplets were incorporated. As a result, large droplets settled on the steps in a few seconds, and small droplets remained suspended for a long time. The dispersion of droplets significantly depended on the traveling direction of the vortex ring generated by a cough jet, due to the effect of downwash or upwash between passengers.

Next, risks of contact with the viral droplets were quantitatively evaluated on the basis of the number of droplets adhering to passenger's heads. The risk mainly increased due to contact with highly concentrated droplets rather than prolonged exposure to droplets around passengers entrained by recirculation or stagnation near steps. Although the risk changed slightly depending on the coughing start time due to unsteady flow fields, the ensemble average showed a clear trend. As expected, a wider passenger spacing reduced the infection risk. The risk was found to be higher for the ascending cases than the descending ones. This was the opposite of the results of Li et al. and Wang et al. where they used simple escalator models with only a single passenger. In summary, physical distancing on an escalator was effective in reducing exposure risk. Because of the droplet transport among the traveling vortex ring, we also found an unexpectedly case: the passenger positioned at a farther distance away from the infected person could present a higher risk than at a closer one. This long‐distance infection was especially remarkable in the ascending case with 10 passengers. We quantitatively estimated the risk of infection using the dose–response model. Notably, the risk of the infection for the second passenger exceeded that for the last passenger by a factor of 25; A passenger near the infected person faces a higher risk of infection. For this reason, the importance of social distancing must be emphasized. In this paper, the isothermal condition was adopted, we regard that this study estimates the risk of infection on the safe side. Incorporating natural convection into the simulation would predict a lower exposure risk because the updraft around a passenger's face keeps away infectious droplets.

Finally, the effect of coughing direction on the rate of droplet adhesion was investigated. We demonstrated that coughing direction has a critical impact on viral droplet adhesion and, as a result, the infected passenger on the escalator is recommended to cough toward the left or right so that following passengers can avoid contact with concentrated droplets expelled. Although the present simulations require large computational resources, numerical simulation is a powerful tool to resolve the strong unsteady nonlinear fluid dynamics. We hope that our paper will motivate the need to assess the risk of viral droplet adhesion via airborne droplets.

## AUTHOR CONTRIBUTIONS

Masashi YAMAKAWA conceived the project. Ayato TAKII performed the numerical simulations and analyzed the data with assistance from Masashi YAMAKAWA, Atsuhide KITAGAWA and Tomoaki WATAMURA. Ayato TAKII wrote the paper. All authors contributed discussing the results and providing feedbacks.

## CONFLICT OF INTEREST

The authors declare that they have no known competing financial interests or personal relationships that could influence the work reported in this paper.

## Supporting information


Appendix S1

Video S1

Video S2

## Data Availability

The data that support the findings of study are available from the corresponding author upon reasonable request.
