# Supplementary material for: Numerical model for cough‐generated droplet dispersion on moving escalator with multiple passengers
Source: Indoor Air. 2022 Nov 18;32(11):e13131. doi: 10.1111/ina.13131 (PMC9827918; doi:10.1111/ina.13131)
Supplement: Supplementary file 1 — Appendix S1 [file INA-32-0-s002.docx]

**Appendix A. Validation of the Moving Computational Domain Method**

To validate the physical conservation law of the moving computational domain method for incompressible flow. The drag coefficient *C_D_* of a sphere in the uniform flow are computed by using the moving grid and compared with results of Shirayama^1^ and Kim et al.^2^. Figure A.1 illustrates the computational conditions. The boundary conditions of velocity are as follows: no-slip at the surface of the sphere, fixed value 0.0 for the inlet, and zero gradient for the outlet. The boundary conditions of pressure are zero gradient for all surfaces. The number of grid cells is 297740, the minimum grid spacing is 0.04, Reynolds number is 100, diameter of a sphere is 1.0. As the initial condition, the velocities in the computational domain is set to 0.0. Based on the MCD method, the entire computational domain is moved through an acceleration of 0.1, after reaching the velocity of 0.1, a constant velocity of 1.0 is maintained. To investigate the dependency on time-stepping, validations were performed at different time-steps $\Delta t$ = 0.001, 0.01, and 0.1. Table A.1 presents a comparison of the value of the converged drag coefficient *C_D_* at *t* = 40, with the results of *C_D_* reported in other studies. The value of *C_D_* for $\Delta t$ = 0.001 and 0.01 agreed with the other results with a difference of less than 1%, as shown in Figure A.2. Therefore, this method satisfies the physical conservation law on the moving grid.

**References**

1. Shirayama, S., “Flow past a sphere : topological transitions of the vorticity fields”, *AIAA Journal* **30(2)**, 349-358 (1992).
2. Kim, D. and Choi, H., “Laminar Flow Past a Sphere Rotating in the Streamwise Direction”, *Journal of Fluid Mechanics* **461**, 365-386 (2002).

5.5

10.5

5.5

Non-Slip Wall

B.C.

Out Flow

B.C.

In Flow B.C.

Moving Direction

**Figure A1** Computational conditions around a sphere for the validation of the moving computational domain method.

**Table A1** Comparison of drag coefficient *C_D_* of the sphere for the validation of the moving computational domain method.

|  | Moving grid (present method) | | | Fixed grid | |
| --- | --- | --- | --- | --- | --- |
|  | $\Delta t=0.1$ | $\Delta t=0.01$ | $\Delta t=0.001$ | Shirayama | Kim et al. |
| $C_{D}$ | 1.154 | 1.105 | 1.092 | 1.104 | 1.087 |

**Figure A2** Computational time step ∆*t* versus error in the drag coefficient |(*C_D_* – *C_D_*^Ref^) / *C_D_*^Ref^ |, where we use the averaged value of the results obtained from Shirayama and Kim et al. *C_D_*^Ref^ = (*C_D_*^Shirayama^ + *C_D_*^Kim^)/2 as the reference data.
