# Supplementary figures and images for: Numerical model for cough‐generated droplet dispersion on moving escalator with multiple passengers
Source: Indoor Air. 2022 Nov 18;32(11):e13131. doi: 10.1111/ina.13131 (PMC9827918; doi:10.1111/ina.13131)

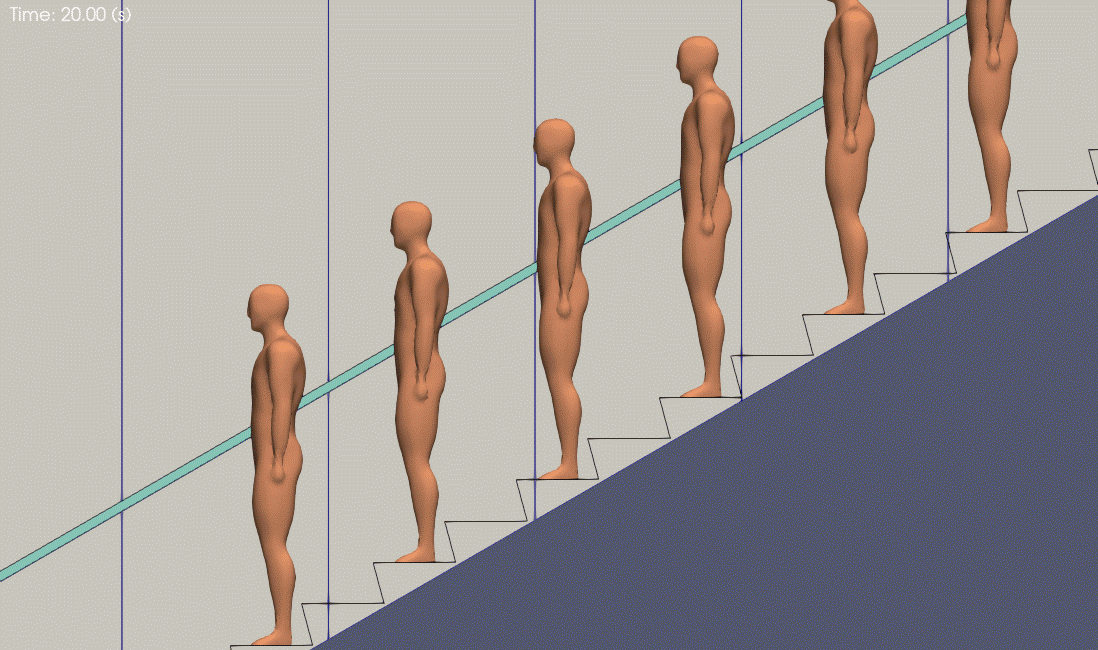

Supplement: Supplementary file 3 — Video S2 [file INA-32-0-s003.gif]
